# Exploring Molecular Mechanisms of Liver Fibrosis

**DOI:** 10.3390/ijms26010326

**Published:** 2025-01-02

**Authors:** Ralf Weiskirchen

**Affiliations:** Institute of Molecular Pathobiochemistry, Experimental Gene Therapy and Clinical Chemistry (IFMPEGKC), University Hospital Aachen, D-52074 Aachen, Germany; rweiskirchen@ukaachen.de

Liver diseases, particularly metabolic dysfunction-associated steatotic liver disease (MASLD), have emerged as a major global health concern, affecting millions of individuals and leading to increased morbidity and mortality [1]. MASLD encompasses a spectrum of conditions ranging from simple hepatic steatosis to more severe forms such as metabolic dysfunction-associated steatohepatitis (MASH), fibrosis, cirrhosis, and hepatocellular carcinoma. The increasing prevalence of obesity, sedentary lifestyles, and unhealthy dietary habits has contributed to the rising incidence of these liver disorders.

Understanding the underlying mechanisms that drive liver injury and disease progression is crucial for developing effective therapeutic strategies. The key factors involved in this process include alterations in lipid metabolism, inflammatory responses, oxidative stress, and gut–liver interactions. Hepatic stellate cells (HSCs) play a central role in fibrogenesis, while various cytokines and signaling pathways influence their activation [2]. Additionally, emerging evidence suggests that the gut microbiota has a significant impact on liver health through mechanisms such as bacterial translocation and the modulation of systemic inflammation [3].

This collection comprises nine contributions (four reviews and five original articles) authored by 65 researchers from six countries (Germany, Republic of Korea, Russia, Serbia, The Netherlands, and the USA) discussing various aspects of liver pathogenesis (Figure 1).

These studies collectively enhance our understanding of the complex interactions between metabolic dysregulation, immune responses, and liver pathology in MASLD. By exploring novel biomarkers associated with liver dysfunction and identifying potential therapeutic targets within these intricate relationships, this body of work aims to improve patient outcomes for those affected by liver diseases. The insights gained will help guide future research efforts focused on addressing the challenges posed by MASLD on a global scale.

In her review, Roeb discusses the multiple roles of interleukin-13 (IL-13) in wound healing and fibrosis, particularly in the context of MASH (Contribution 1). The involvement of the liver in systemic metabolic-inflammatory diseases is highlighted, emphasizing how IL-13 can exert both protective and detrimental effects. While IL-13 can promote metabolic functions and Th2-polarized inflammation, it is also associated with the loss of intestinal barrier function and increased liver fibrosis, complicating its therapeutic potential. The author discusses the increasing prevalence of MASH worldwide, underscoring the urgent need for effective pharmacotherapies. Despite the recognized involvement of IL-13 in various inflammatory processes, research into its specific impact on metabolic diseases remains limited. The author reviews the contrasting findings regarding the role of IL-13; while it can promote wound healing and positively regulate immune responses, excessive IL-13 signaling can lead to adverse outcomes such as fibrosis and impaired barrier integrity. In addition, the review synthesizes the recent literature on the signaling pathways and cellular interactions of IL-13 in different tissues. It is suggested that understanding these complex mechanisms could inform novel therapeutic strategies targeting IL-13 for the treatment of MASH and related conditions. In conclusion, Roeb advocates for further investigation into the dual nature of IL-13 as a cytokine involved in both promoting tissue repair and contributing to pathological fibrosis. This nuanced understanding may pave the way for innovative treatments aimed at mitigating liver damage while harnessing the beneficial aspects of this pleiotropic cytokine.

In their review, Maslennikov et al. examine the critical role of the gut microbiota and bacterial translocation in the pathogenesis of liver fibrosis, particularly in chronic liver diseases leading to cirrhosis (Contribution 2). The authors highlight that intestinal dysbiosis, characterized by an increase in harmful bacteria from the Bacilli class and the *Proteobacteria* phylum, alongside a decrease in beneficial *Clostridia*, occurs even before the onset of cirrhosis. This imbalance weakens the intestinal barrier, facilitating bacterial translocation into the bloodstream, which subsequently activates Kupffer cells in the liver via Toll-like receptor 4 (TLR4). Activated Kupffer cells release profibrogenic cytokines that stimulate HSCs to transform into collagen-producing myofibroblasts, driving liver fibrosis. The review emphasizes that targeting bacterial translocation using antibiotics, probiotics, and synbiotics may help slow down liver fibrosis progression, as demonstrated in various animal models. However, further long-term randomized controlled trials (RCTs) are needed to validate these findings in humans. Additionally, the authors discuss how chronic inflammation resulting from bacterial translocation contributes to ongoing liver damage and fibrosis, even after addressing underlying causes such as alcohol consumption or viral infections. They conclude that understanding and intervening at the gut–liver axis may provide new therapeutic avenues to prevent or alleviate liver fibrosis in patients with chronic liver disease.

Chandrasekaran and Weiskirchen reviewed the critical role of sterol regulatory element-binding proteins (SREBPs) and SREBP cleavage activating protein (SCAP) in lipid metabolism and the pathogenesis of MASLD (Contribution 3). The increasing prevalence of MASLD is attributed to obesity and unhealthy lifestyles, leading to a spectrum of liver diseases ranging from steatosis to hepatocellular carcinoma. SREBP1c has been identified as a key transcription factor regulating hepatic de novo lipogenesis, which is essential for fatty acid synthesis. The review highlights how SCAP acts as a chaperone for SREBP, facilitating its transport from the endoplasmic reticulum to the Golgi apparatus for proteolytic activation. When cholesterol levels are high, SCAP binds to INSIG proteins, preventing SREBP activation and thus inhibiting lipid synthesis. Conversely, low cholesterol levels promote SREBP activity, thereby enhancing lipogenesis. The authors discuss several regulatory mechanisms that influence SCAP/SREBP function, including insulin signaling and feedback loops that maintain lipid homeostasis. They highlight the fact that targeting the SCAP/SREBP axis may offer therapeutic opportunities for the management of MASLD. Recent studies suggest that the inhibition of this pathway may resolve hepatic steatosis in animal models. In conclusion, understanding the intricate roles of SCAP and SREBP in lipid metabolism provides insight into potential interventions for MASLD. The authors advocate for further research into pharmacological agents targeting this axis as a promising therapeutic strategy against liver diseases associated with metabolic dysfunction.

Vesković et al. review the pathophysiological mechanisms of liver fibrosis in lean versus obese metabolic-associated fatty liver disease (MAFLD), highlighting the different characteristics and progression of fibrosis in these two populations (Contribution 4). While obesity-related non-alcoholic fatty liver disease (NAFLD) is commonly associated with metabolic abnormalities such as insulin resistance and inflammation, lean NAFLD can progress to fibrosis without these typical risk factors, often due to genetic predispositions or changes in the gut microbiota. The authors summarize key findings from several animal models used to study lean NAFLD, including methionine/choline-deficient (MCD) diets and high-fructose diets, which reveal significant pro-fibrogenic mechanisms such as the increased activation of the extracellular signal-regulated kinase (ERK) pathway and the increased expression of fibrogenic markers such as transforming growth factor-β (TGF-β) and α-smooth muscle actin (α-SMA). They highlight that although both lean and obese NAFLD are associated with fibrosis, the underlying molecular pathways may differ significantly, requiring tailored therapeutic approaches. In addition, the review underscores the importance of targeting the specific pathways involved in fibrosis development to identify novel biomarkers for the early detection and management of lean NAFLD. The authors advocate for further research into targeted therapies that could improve clinical outcomes for patients suffering from this increasingly prevalent condition. Overall, this comprehensive comparison enhances our understanding of the pathogenesis of fibrosis in MAFLD and highlights critical avenues for future investigation aimed at effective treatment strategies.

In their original article, Hwang et al. investigate the role of Krüppel-like factor 10 (KLF10) in inhibiting the activation of HSCs mediated by TGF-β, focusing on its regulatory effects on activating transcription factor 3 (ATF3) (Contribution 5). Liver fibrosis is a major health problem characterized by excessive extracellular matrix deposition, driven primarily by activated HSCs in response to TGF-β signaling. The article begins by showing that KLF10 is induced early after TGF-β treatment, while ATF3 expression shows a delayed response. Using the RNA sequencing of liver tissue from KLF10 knockout mice that were fed a high-sucrose diet, the authors identify ATF3 as a potential target gene regulated by KLF10. They show that KLF10 knockdown enhances the TGF-β-mediated activation of the immortalized human HSC line LX-2, leading to increased levels of fibrogenic proteins and collagen deposition. Mechanistic studies reveal that KLF10 knockdown promotes TGF-β signaling and upregulates ATF3 expression, whereas KLF10 overexpression has the opposite effect. Furthermore, treatment with the chemical chaperone 4-PBA attenuates the siKLF10-induced upregulation of ATF3 and fibrogenic responses in TGF-β-treated LX-2 cells. These findings suggest that KLF10 acts as a negative regulator of TGF-β signaling and highlight its potential as a therapeutic target for liver fibrosis through the modulation of the KLF10-ATF3 axis. Overall, this research provides valuable insights into the molecular mechanisms underlying HSC activation and offers promising avenues for the development of targeted therapies for liver fibrosis.

Schilcher et al. investigate the role of saturated fatty acids in the upregulation of interleukin-32 (IL-32) and chemokine CC ligand 20 (CCL20) in hepatocytes, contributing to fibrosis in MASLD (Contribution 6). The study highlights that MASLD encompasses a spectrum of liver diseases, ranging from simple steatosis to more severe conditions such as MASH, which can progress to cirrhosis and hepatocellular carcinoma. The authors report that IL-32 and CCL20 expression is significantly increased in liver tissue from patients with MASH compared to normal tissue. They show that treatment with palmitic acid (PA), a saturated fatty acid, induces the expression of both IL-32 and CCL20 in human hepatoma cell lines HepG2 and Huh7. This induction is attenuated by oleic acid (OA), a monounsaturated fatty acid, suggesting a protective effect against PA-induced inflammation. Mechanistic studies reveal that the upregulation of IL-32 and CCL20 by PA is mediated by stress-induced signaling pathways involving ERK1/2 and p38 MAPK. The findings suggest that saturated fat contributes to liver inflammation and fibrosis through the activation of these pro-inflammatory molecules. Overall, this research underscores the importance of understanding how dietary components influence the development of hepatic inflammation and fibrosis in MASLD. Targeting IL-32 and CCL20 may provide new therapeutic avenues for managing the progression of liver disease associated with metabolic dysfunction.

Efremova et al. present data investigating the relationship between gut microbiota, biomarkers of endothelial dysfunction, and manifestations of cirrhosis in patients with MASLD (Contribution 7). The study highlights the importance of understanding how changes in the gut microbiota contribute to systemic inflammation and vascular complications associated with liver disease. The researchers performed 16S rRNA gene sequencing on fecal samples from 47 cirrhotic patients and 27 healthy controls to assess the composition of the gut microbiota. They measured plasma levels of biomarkers associated with endothelial dysfunction, including nitrites, asymmetric dimethylarginine (ADMA), big endothelin-1, and claudin 3. They found that the levels of nitrite, ADMA, and presepsin were significantly elevated in cirrhotic patients compared to controls. Notably, high levels of nitrite correlated positively with the abundance of harmful *Proteobacteria* and inversely with beneficial taxa such as *Oscillospiraceae*. In addition, the study found that ADMA levels were associated with the clinical features of cirrhosis severity and hemodynamic parameters. The authors suggest that increased intestinal permeability due to dysbiosis may facilitate bacterial translocation into circulation, leading to endothelial dysfunction and contributing to portal hypertension. Overall, this research provides insight into the interplay between gut health and liver pathology in MASLD. It highlights the need for further studies focusing on gut microbiota modulation as a potential therapeutic strategy to treat liver fibrosis and its complications in cirrhosis patients.

In their original article, Gross et al. investigate the role of amyloid beta (Aβ) metabolism in the context of hepatic steatosis and MASH (Contribution 8). The study focuses on how saturated fatty acids, particularly PA, influence the expression of amyloid precursor protein (APP) and its associated metabolizing proteins in hepatocytes, thereby contributing to liver pathology. The authors show that treatment with PA significantly increases the mRNA levels of APP and key Aβ-metabolizing proteins in human hepatoma cell lines and primary human hepatocytes. This effect is attenuated by OA, suggesting a protective role against saturated fat-induced cellular stress. In vivo studies using a high-fat diet mouse model show similar patterns, with an increased expression of APP and neprilysin but unchanged Aβ-42 levels in steatotic livers, indicating altered lipid metabolism without affecting hepatic Aβ-42 concentrations. Furthermore, the study highlights reduced hepatic Aβ-42 levels in patients with MASH compared to those with simple steatosis or healthy controls. The authors note that this reduction correlates with an increased expression of genes involved in non-amyloidogenic pathways, such as ADAM9/10/17 and BACE2, suggesting a shift towards processes that reduce Aβ production. Collectively, these findings emphasize the complex interplay between lipid metabolism and Aβ processing in the liver and provide insights into potential therapeutic targets for the treatment of MASLD. By elucidating these mechanisms, the study contributes to a better understanding of how dietary components can influence liver health and disease progression.

Li et al. investigate the relationship between circulating citrate levels and all-cause mortality in patients with end-stage liver disease (ESLD) (Contribution 9). The study highlights the potential of plasma citrate as a biomarker of mitochondrial dysfunction and its association with disease severity in ESLD patients awaiting liver transplantation. The authors performed a comparative analysis of plasma citrate levels in 129 ESLD patients and 4837 participants from a control cohort. They found that plasma citrate concentrations were significantly elevated in ESLD patients by approximately 40% compared to controls. Notably, in a subset of patients that were followed after liver transplantation, citrate levels decreased to levels below those observed in controls, suggesting reversibility after successful surgical intervention. Further analysis revealed significant correlations between elevated citrate levels and clinical parameters such as Child–Turcotte-Pugh classification and estimated glomerular filtration rate (eGFR). Importantly, higher citrate tertiles were associated with an increased risk of all-cause mortality, particularly in male patients. The findings underscore the importance of monitoring plasma citrate as it may reflect underlying metabolic abnormalities associated with poor outcomes in ESLD. The study suggests that elevated circulating citrate may serve as a useful prognostic marker of mortality risk in this patient population. Overall, this research provides insight into the role of metabolic factors in liver disease progression and emphasizes the need for the further exploration of potential therapeutic strategies targeting metabolic dysregulation in the management of ESLD.

In summary, the nine studies included in this Special Issue collectively enhance our understanding of the complex interplay between metabolic dysfunction, liver pathology, and systemic health. One study elucidates the critical role of a specific transcription factor in regulating the TGF-β-mediated activation of hepatic stellate cells through the modulation of a key stress-responsive protein, highlighting potential therapeutic avenues for liver fibrosis. Another study shows how saturated fatty acids induce the expression of pro-inflammatory cytokines in hepatocytes, contributing to inflammation and fibrogenesis in MASLD. In addition, findings show that elevated circulating citrate levels in patients with end-stage liver disease are associated with an increased risk of mortality, particularly in men, highlighting the importance of metabolic biomarkers in predicting outcome.

Moreover, these contributions underscore the importance of targeting specific molecular pathways and biomarkers to improve clinical management and therapeutic strategies for liver disease. The findings support further research into the mechanisms underlying these processes and their implications for patient care in metabolic liver disease. By improving our understanding of these interactions, there is potential to develop innovative interventions aimed at mitigating liver injury and improving patient prognosis in different clinical scenarios.

## Figures and Tables

**Figure 1:**
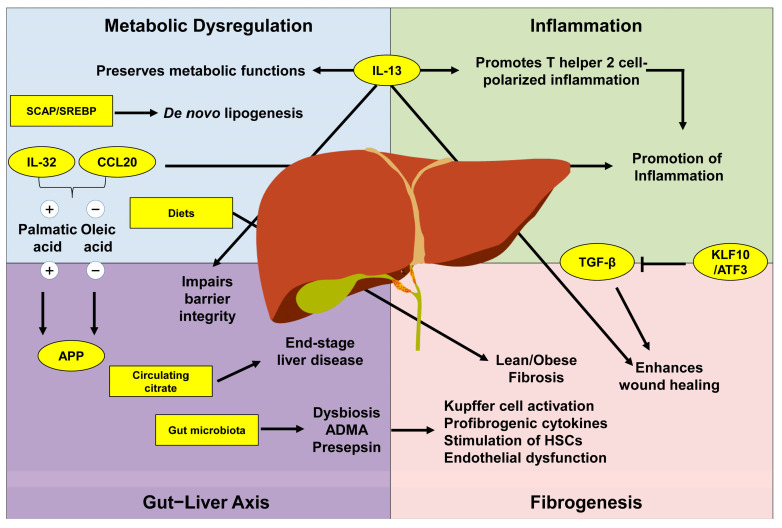
Factors driving liver pathogenesis. Metabolic dysregulation, inflammation, fibrogenesis, and changes in the gut−liver axis can contribute to the pathogenesis of liver disease. This Special Issue contains nine articles discussing various aspects of disease progression. For more details, please refer to the text.
